# Supplementary material for: The Consequences of the Pandemic on Medical Students’ Depressive Symptoms and Perceived Stress: A Repeated Cross-Sectional Survey with a Nested Longitudinal Subsample
Source: J Clin Med. 2022 Oct 6;11(19):5896. doi: 10.3390/jcm11195896 (PMC9572955; doi:10.3390/jcm11195896)
Supplement: Supplementary file 1 [file jcm-11-05896-s001.zip › jcm-1931847-supplementary.pdf]

Supplemental Tables

Table S1. Changes from the first timepoint to the second timepoint (Study 1)

**Characteristic**		**Overall descriptive analysis (n=121)**	**BDI-score**		**PSS-10 score**	
		**N (%)**	**Median (IQR)**	**p-value**	**Mean**	**p-value**
Living condition	Unchanged	112 (94.1)	14 (7-22)	0.247	22 (8.1)	0.967
	Changed in “living alone”	3 (2.5)	16 (14-18)		22 (4.7)	
	Changed in “not living alone”	4 (3.4)	7 (3-13)		21 (7.4)	
Family cohesion	Unchanged	109 (90.1)	13 (7-21)	0.073	21 (7.8)	**0.004**
	Worsened	5 (4.1)	19 (17-19)		27 (4.0)	
	Improved	7 (5.8)	31 (13-37)		30 (6.2)	
Relationship status	Unchanged	91 (75.8)	15 (7-21)	0.842	21 (7.9)	0.800
	Changed in “no relationship”	12 (10.0)	12 (8-19)		23 (7.9)	
	Changed into a relationship	17 (14.2)	13 (8-23)		22 (8.2)	
Sexual orientation	Unchanged	98 (83.1)	14 (7-21)	0.639	22 (7.9)	0.139
	Changed answer	20 (16.9)	14 (8-25)		24 (6.6)	
Economic situation	Unchanged	113 (94.2)	13 (7-21)	0.510	22 (7.9)	0.628
	Worsened	6 (5.0)	20 (13-26)		24 (9.7)	
	Improved	1 (0.8)	16		28	
Judgement on medical school choice	Unchanged	90 (75.0)	12 (7-18)	**<0.001**	21 (7.8)	**<0.001**
	Worsened	24 (20.0)	27 (19-32)		27 (6.0)	
	Improved	6 (5.0)	7 (6-11)		15 (9.0)	
Friendships with classmates	Unchanged	106 (91.4)	13 (7-21)	0.068	22 (8.2)	0.127
	Worsened	9 (7.8)	21 (17-22)		26 (4.4)	
	Improved	1 (0.9)	4		13	
Judgment on climate among classmates	Unchanged	98 (81.7)	12 (6-21)	**0.043**	21 (8.2)	0.059
	Worsened	21 (17.5)	17 (15-25)		26 (5.6)	
	Improved	1 (0.8)	23		27	

Overall descriptive analyses: figures are expressed as frequencies and column percentages in brackets.
BDI-II: figures are expressed as medians and interquartile range in brackets. P-value obtained via Kruskall Wallis test (except for sexual orientation: Mann Whitney U test)
PSS-10: figures are expressed as mean and standard deviation in brackets. P-value obtained via one-way analysis of variance (ANOVA) (except for sexual orientation: t-test)
Abbreviations: BDI-II Beck Depression Inventory II; IQR Interquartile Range; PSS-10 Perceived Stress Scale 10; SD Standard Deviation.

Table S2. Variables belonging to the second timepoint survey: descriptive analysis and outcomes' distribution (Study 1)

Characteristic (Timepoint 2)		Descriptive analysis	BDI-II (timepoint 2)		PSS-10 (timepoint 2)	
			Median (IQR)	p-value	Mean (SD)	p-value
Poor quality of relations with house cohabitants	No	108 (90.8)	13 (7-21)	**0.016**	21.5 (7.7)	**0.041**
	Yes	11 (9.2)	21 (13-37)		26.6 (9.7)	
Poor quality of relations with house cohabitants during lockdown	No	109 (91.6)	13 (7-21)	**0.009**	21.3 (7.8)	**0.004**
	Yes	10 (8.4)	20 (13-37)		28.8 (6.0)	
Availability of common spaces in the house	No	5 (4.2)	13 (4-22)	0.867	22.2 (10.8)	0.949
	Yes	114 (95.8)	14 (7-21)		22.0 (7.9)	
Fear of COVID-19 contagion for loved ones	No	22 (18.6)	17 (12-32)	**0.018**	24.1 (9.1)	0.168
	Yes	96 (81.4)	13 (7-21)		21.5 (7.6)	
Loved one vulnerable/ at risk for COVID-19	No	13 (10.8)	17 (11-31)	0.130	25.4 (7.3)	0.102
	Yes	107 (89.2)	13 (7-21)		21.6 (8.0)	
Economic repercussions due to COVID-19	No	98 (83.1)	13 (7-20)	0.053	21.3 (7.9)	**0.032**
	Yes	20 (17.0)	19 (11-25)		25.5 (7.6)	
Feeling of loneliness during pandemic	No	31 (26.3)	8 (5-18)	**0.027**	18.0 (7.1)	**0.003**
	Yes, more than usual	74 (62.7)	16 (9-22)		23.2 (7.6)	
	Yes, as usual	13 (11.0)	21 (11-26)		24.7 (9.3)	
Existential reflections related to COVID-19	No	28 (23.7)	13 (8-18)	**<0.001**	20.5 (8.9)	**0.002**
	Yes, positively stimulating	43 (36.4)	9 (5-17)		19.5 (7.6)	
	Yes, deeply distressing	47 (39.8)	20 (13-29)		25.1 (6.7)	
Overall psychological condition changed due to COVID-19	No	49 (41.5)	8 (5-17)	**<0.001**	18.1 (7.2)	**<0.001**
	Yes, improved	3 (2.5)	5 (1-22)		23.0 (9.6)	
	Yes, worsened	66 (55.9)	18 (13-27)		24.8 (7.3)	

Descriptive analysis: figures are expressed as frequencies and column percentages in brackets.
BDI-II: Figures are expressed as median and interquartile range in brackets. P-value obtained via Mann Whitney U test and Kruskal Wallis test.
PSS-10: Figures are expressed as mean and standard deviation in brackets. P-value obtained via t-test and one-way analysis of variance (ANOVA).
Abbreviations: BDI-II Beck Depression Inventory II; IQR Interquartile Range; PSS-10 Perceived Stress Scale 10; SD Standard Deviation.

Table S3. Distribution of BDI-II and PSS-10 scores across the independent variables in the two timepoints (Study 1)

Characteristic		BDI-II				PSS-10			
		First timepoint<br><br>Median (IQR)	p-value	Second timepoint<br><br>Median (IQR)	p-value	First timepoint<br><br>Median	p-value	Second timepoint<br><br>Median	p-value
Gender	Male	8 (4-11)	0.868	18 (9-24)	0.119	15.4 (7.3)	0.316	21.8 (8.1)	0.899
	Female	7 (3-13)		13 (7-19)		16.8 (7.2)		22.0 (8.0)	
Off-site student	No	7 (3-13)	0.936	16 (7-22)	0.603	15.7 (7.3)	0.258	21.6 (7.8)	0.604
	Yes	8 (4-11)		13 (7-21)		17.2 (7.2)		22.3 (8.2)	
Living alone	No	7 (3-12)	0.393	13 (7-12)	0.579	16.2 (7.2)	0.336	21.9 (8.1)	0.814
	Yes	6 (1-10)		16 (14-18)		19.2 (6.7)		22.8 (3.7)	
Very poor/poor family cohesion	No	7 (3-11)	0.259	13 (7-21)	**0.016**	16.2 (7.1)	0.375	21.1 (7.7)	**0.001**
	Yes	8 (4-17)		21 (13-32)		18.1 (8.1)		28.2 (6.8)	
Relationship status	Single	6 (3-11)	0.287	14 (7-23)	0.781	16.3 (7.0)	0.841	21.9 (7.6)	0.882
	Involved	8 (3-13)		13 (8-21)		16.5 (7.6)		22.1 (8.4)	
LGBA sexual orientation	No	7 (3-11)	**0.020**	13 (7-21)	0.095	16.1 (7.5)	0.328	21.2 (7.7)	0.078
	Yes	11 (5-15)		17 (11-25)		17.8 (6.1)		24.1 (8.3)	
Family history of psychiatric disorders	No	6 (2-11)	**0.002**	12 (7-20)	0.112	15.0 (6.9)	**<0.001**	21.4 (8.0)	0.140
	Yes	10 (6-14)		17 (12-23)		20.2 (6.6)		23.4 (7.6)	
Very poor/poor economic status	No	7 (3-11)	0.145	13 (7-22)	0.295	16.2 (7.2)	0.367	21.7 (8.0)	0.081
	Yes	9 (6-16)		19 (16-22)		18.3 (7.0)		27.5 (6.3)	
Negative judgement of medical school choice	No	7 (3-11)	0.773	11 (6-17)	**<0.001**	16.0 (7.2)	0.080	20.5 (8.1)	**<0.001**
	Yes	6 (4-14)		23 (19-30)		19.7 (6.3)		26.6 (5.6)	
Unsatisfying friendships with classmates	No	8 (3-12)	0.678	13 (7-21)	0.059	16.4 (7.2)	-	21.6 (8.2)	0.081
	Yes	5 (5-5)		21 (17-22)		-		26.4 (4.4)	
Climate among classmates judged as hostile/competitive	No	7 (3-11)	**0.017**	12 (6-21)	**0.008**	16.2 (7.0)	0.064	21.1 (8.2)	**0.011**
	Yes	35 (27-42)		17 (15-25)		24.0 (13.5)		25.7 (5.7)	

BDI-II: Figures are expressed as median and interquartile range in brackets. P-value obtained via Mann Whitney U test.
PSS-10: Figures are expressed as mean and standard deviation in brackets. P-value obtained via t-test.
Abbreviations: BDI-II Beck Depression Inventory II; IQR Interquartile Range; LGBA Lesbian Gay Bisexual Asexual; PSS-10 Perceived Stress Scale 10; SD Standard Deviation.

Table S4. Distribution of BDI-II and PSS-score across the independent variables (Study 2)

Characteristic		BDI-score		PSS-10 score	
		**Median (IQR)**	**p-value**	**Median (IQR)**	**p-value**
Gender	Male	9 (5-16)	**<0.001**	18 (12-23)	**<0.001**
	Female	13 (8-21)		23 (17-27)	
Year of course	4th	12 (7-20)	0.248	22 (16-27)	0.381
	6th	12 (6-19)		21 (15-26)	
Off-site student	No	12 (7-19)	0.999	21 (16-27)	0.688
	Yes	12 (6-20)		21 (15-27)	
Living alone	No	12 (7-19)	0.183	22 (16-27)	0.060
	Yes	9 (5-17)		16 (12-26)	
Very poor/poor family cohesion	No	11 (6-18)	**<0.001**	21 (15-26)	**<0.001**
	Yes	17 (11-24)		24 (20-29)	
Relationship status	Single	12 (7-21)	0.299	21 (15-26)	0.759
	Involved	12 (7-19)		21 (15-27)	
LGBA sexual orientation	No	12 (6-19)	**0.012**	21 (15-26)	**0.005**
	Yes	14 (8-22)		23 (17-28)	
Family history of psychiatric disorders	No	12 (6-19)	0.052	21 (15-27)	0.288
	Yes	13 (7-21)		22 (16-28)	
Very poor/poor economic status	No	12 (6-19)	**0.010**	21 (15-27)	**0.003**
	Yes	16 (11-22)		24 (21-28)	
Negative judgement of medical school choice	No	10 (5-15)	**<0.001**	19 (13-24)	**<0.001**
	Yes	18 (23-26)		25 (20-29)	
Unsatisfying friendships with classmates	No	11 (6-18)	**<0.001**	21 (15-26)	**<0.001**
	Yes	21 (10-29)		25 (19-30)	
Climate among classmates judged as hostile/competitive	No	11 (6-18)	**<0.001**	21 (15-26)	**0.002**
	Yes	15 (10-23)		23 (17-29)	

Figures are expressed as medians and interquartile range in brackets. P-value obtained via Mann Whitney U test.
Abbreviations: BDI-II Beck Depression Inventory II; IQR Interquartile Range; LGBA Lesbian Gay Bisexual Asexual; PSS-10 Perceived Stress Scale 10.
